# Chronic Perigestational Exposure to Chlorpyrifos Induces Perturbations in Gut Bacteria and Glucose and Lipid Markers in Female Rats and Their Offspring

**DOI:** 10.3390/toxics10030138

**Published:** 2022-03-12

**Authors:** Narimane Djekkoun, Flore Depeint, Marion Guibourdenche, Hiba El Khayat El Sabbouri, Aurélie Corona, Larbi Rhazi, Jerome Gay-Queheillard, Leila Rouabah, Farida Hamdad, Véronique Bach, Moncef Benkhalifa, Hafida Khorsi-Cauet

**Affiliations:** 1 PeriTox UMR_I 01 Laboratory, University Center for Health Research, CURS-UPJV, Picardy Jules Verne University, CEDEX 1, 80054 Amiens, France; djekkoun.narimane@gmail.com (N.D.); marion.guibourdenche@outlook.fr (M.G.); hiba.el-khayat-el-sabbouri@univ-cotedazur.fr (H.E.K.E.S.); aurelie.corona@u-picardie.fr (A.C.); jerome.gay@u-picardie.fr (J.G.-Q.); veronique.bach@u-picardie.fr (V.B.); benkhalifamoncef78@gmail.com (M.B.); 2 Laboratory of Cellular and Molecular Biology, University of the Brothers Mentouri Constantine 1, Constantine 2500, Algeria; leilarouabah27@yahoo.fr; 3 Transformations & Agro-Ressources ULR7519, Institut Polytechnique UniLaSalle—Université d’Artois, 60026 Beauvais, France; flore.depeint@unilasalle.fr (F.D.); larbi.rhazi@unilasalle.fr (L.R.); 4 Center for Human Biology, CHU Amiens-Picardie, 80000 Amiens, France; hamdadfarida2002@yahoo.fr

**Keywords:** pesticides, prebiotic, intestinal dysbiosis, perigestational, dysmetabolism, risk factor

## Abstract

An increasing burden of evidence is pointing toward pesticides as risk factors for chronic disorders such as obesity and type 2 diabetes, leading to metabolic syndrome. Our objective was to assess the impact of chlorpyrifos (CPF) on metabolic and bacteriologic markers. Female rats were exposed before and during gestation and during lactation to CPF (1 mg/kg/day). Outcomes such as weight, glucose and lipid profiles, as well as disturbances in selected gut bacterial levels, were measured in both the dams (at the end of the lactation period) and in their female offspring at early adulthood (60 days of age). The results show that the weight of CPF dams were lower compared to the other groups, accompanied by an imbalance in blood glucose and lipid markers, and selected gut bacteria. Intra-uterine growth retardation, as well as metabolic disturbances and perturbation of selected gut bacteria, were also observed in their offspring, indicating both a direct effect on the dams and an indirect effect of CPF on the female offspring. Co-treatment with inulin (a prebiotic) prevented some of the outcomes of the pesticide. Further investigations could help better understand if those perturbations mimic or potentiate nutritional risk factors for metabolic syndrome through high fat diet.

## 1. Introduction

Chemical pollution of the environment by insecticides has become a global phenomenon [1]. They are defined as chemicals used to prevent and control pests, including vectors of human or animal diseases. They are used to control unwanted plant or animal species that interfere with agricultural products [2,3,4]. Pesticides can include herbicides, insecticides, fungicides, disinfectants and rodenticides [3]. According to the most recent statistics on agriculture, forestry and fisheries for the European Union, the total quantity of pesticides sold in Europe amounted to almost 360,000 tons with a significant use of fungicides and bactericides (44%), and herbicides (32%) [5].

The use of pesticides in both developing and developed countries has increased dramatically in recent times. They are widely used in agriculture to increase the yields, quality and appearance of products and reduce the need for agricultural labor [6]. Thus, the potential contamination of the environment by pesticides raises concerns for the public and regulators. In the process of reducing phytochemical products, France has implemented National Health and Environment Plans (PNSE1: 2004–2008, PNSE2: 2009–2013, PNSE3: 2015–2019, PNSE 4: 2020–2024), initiated by the law of 9 August 2004 relating to public health policy. They aim to study the health consequences of exposure to various environmental pollution and include, among other things, estimating the population’s exposure to pesticides, improving knowledge of pesticide exposure and the monitoring of occupational exposures. The Ecophyto plan was created in 2008 to reduce the use of phytosanitary products in France whilst maintaining economically efficient agriculture. Initially, the Ecophyto plan aimed to reduce the use of these substances by 50% by 2020. The target has been extended to 2025 in the face of implementation difficulties [7,8].

Some pesticides are considered endocrine disruptors [9]; therefore, daily exposure is likely to have serious and irreversible effects on the health of individuals [10]. Organophosphates (OP) represent the largest category used in the world and the most widespread due to their bioaccumulation in the environment [11,12,13,14], although their use in France is currently declining due to their consequences on animal and human health. Human exposure to these pesticides is mainly oral, by ingestion of pesticide residues in fruits and vegetables [15]. The extensive and indiscriminate use of OP pesticides in agriculture has been of major concern due to its potentially known or suspected harmful effects on humans. Among the most widely used OP is chlorpyrifos (CPF). Chlorpyrifos was first synthesized by German researchers in 1930 and first introduced in the United States in 1965 as a household insecticide by The Dow Chemical Company (Midland, MI, USA) [16,17]. According to the classification of the World Health Organization (WHO), chlorpyrifos is a class II pesticide of moderate toxicity [18]. CPF is a potent OP insecticide with low water solubility (80.9 mg/L) and a high adsorption coefficient in soil, has a longer half-life in soil (ranging from 65 to 360 days) [19], a wide spectrum of insecticidal action and relative safety compared to other organophosphates, which has led to its intensive use in agriculture. After the application of chlorpyrifos, less than 0.1% of the pesticides applied have a real impact on the intended target. The rest of the residue remains in the environment [16].

CPF use has been closely monitored in recent years, and while it is officially still authorized in 20 EU member states, renewal has been postponed pending further safety reports. The UK government adopted strict restrictions on the use of chlorpyrifos in 2016 due to new safety issues for human health. In the United States, the use of chlorpyrifos indoors has been banned since 2001. The ban process for agricultural use began in 2015in California, and Hawaii banned the sale and use of chlorpyrifos at the end of 2020 [20]. However, the situation remains unclear and CPF can still be found in a number of settings.

CPF is recognized as a neurotoxic agent due to its inhibitory effect on various cholinesterase (ChE) enzymes at the central nervous (CNS) and systemic level [21]. High levels of pesticide residues have been found in several human cohorts [22,23,24,25,26]. According to the United States Environmental Protection Agency (EPA), the no-observed-adverse-effect level (NOAEL) of CPF for acute dietary exposure for the inhibition of red blood cell cholinesterase is 0.5 mg/kg/day [27]. Exposure to CPF has been linked to significant alterations in metabolites that interfere with cellular energy production and amino acid metabolism [28,29,30]; it also acts on hormonal signaling [31,32] and metabolism [33,34], and leads to the disruption of glucose and lipid metabolisms [35,36,37,38], leading to weight gain [39,40,41,42] and increasing the risk of developing chronic non-communicable diseases.

Considering that the oral route is the main cause of human exposure, the impact of a pesticide on the digestive tract is of particular interest. Since it is the first physiological barrier to come into contact with ingested food contaminants, many studies began to focus on the impact of CPF on the gut barrier and gut microbiota [35,43,44,45,46]. The gut microbiota refers to billions of microorganisms residing in the intestine and has a mutualistic relationship with its host [47]. It has several functional roles and impacts on human physiology. It modulates the host’s nutrition through the production of some vitamins and the fermentation of non-digestible food components by the host, protects against pathogens [48] and drug metabolism, and influences intestinal epithelial homeostasis [49].

Exposure to CPF induced by disturbance is often characterized by a decrease in the number of beneficial microorganisms and a simultaneous increase in the number of potentially pathogenic microorganisms leading to dysbiosis [50,51,52,53]. In addition, CPF has been shown to increase intestinal permeability in rats [43,45] or in vitro [54,55], inducing a bacterial translocation which corresponds to the passage of viable bacteria of the gastrointestinal flora through the barrier of the intestinal mucosa (the lamina propria), to the mesenteric nodes, then to normally sterile internal organs such as the spleen and the liver [56].

Pregnancy is a very sensitive period of life where epigenetic marks can impact the long-term development of chronic disorders in the next generation. This is known as the concept of “developmental origin of health and disease” (DOHaD). Contamination of CPF during this period can lead to delayed maturation of the gut microbiota, affecting the bowel function [57]. This may contribute to the onset of obesity and type 2 diabetes (T2D) later in life [58].

Compelling evidence suggests that oral prebiotic supplementation improves these metabolic disorders [59,60]. Additionally, prebiotics are likely associated with increased bifidobacteria and lactobacilli, and the production of short chain fatty acids (SCFA), which are involved in modulating host metabolism [61]. They also strengthen the intestinal barrier, increase satiety by promoting intestinal hormones, improve glucose tolerance, and counteract fatty liver disease (lipogenesis) and insulin resistance [62].

The main objective of the study was to evaluate the effect of perigestional exposure to CPF on the metabolic regulations of dams and their female offspring in early adulthood. For this purpose, weight, lipid and glucose metabolism, levels of selected intestinal bacteria and bacterial translocation were assessed. The secondary objective was to study the protective effect of a prebiotic (inulin) on the same parameters.

## 2. Materials and Methods

### 2.1. Experimental Conditions

Chlorpyrifos (*O*,*O*-diethyl-*O*-(3,5,6-trichloro-2-pyridinyl)-phosphorothioate) with a purity of 99.8% was supplied by LGC Standards (Molsheim, France). Inulin was a kind gift from Cosucra (Belgium). Wistar rats were purchased from Janvier laboratories (Le Genest Saint Isle, France). Animal standard diet (Serlab3436, 3.1 kcal/g, Serlab, Montataire, France) was identical throughout the study.

The experiment was carried out according to the protocol approved by the Regional Directorate for Health, Animal Protection and the Environment (Amiens, France) and the Ministry of Research (reference number APAFIS # 8207-2016121322563594 v2 approved on 5 September 2017). All animals were treated in accordance with the EU Directive 2010/63.

Animals were housed in a NexGen Max cage system mounted on an EcoFlow rack system (Allentown Inc, Bussy Saint Georges, France) under constant conditions in a temperature- and air-controlled room (23 °C) with a 12-h light/dark cycle. The size of each group was set to four females for the reproduction protocol in order to reach a minimum of 6–10 female offspring for the passive impact analysis. After acclimation, sixteen 7-week old female Wistar rats (body weight (b.w.) 225 ± 4.9 g) were randomly assigned to four groups (*n* = 4/group) and housed two per cage.

**Figure 1:**
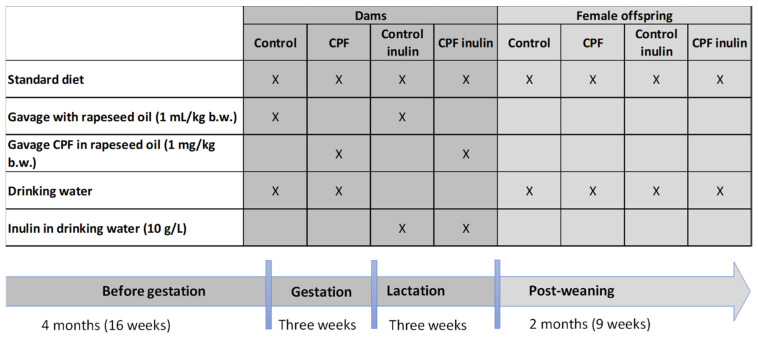
Treatment groups. Dams were fed standard diet and various treatments per os before gestation until weaning at which time they were sacrificed. Their female offspring were fed a standard diet only until 60 days of age at which time they were sacrificed. CPF: Chlorpyrifos; b.w.: body weight.

Chlorpyrifos was dissolved in commercially available organic rapeseed oil at a concentration of 10 mg/mL. CPF solution or rapeseed oil only were administered daily (5 consecutive days followed by a 2-day break) by gavage at a dose of 1 mL/kg b.w. to the animals. This was equivalent to a final concentration of 1 mg/kg b.w. (the oral “no observed adverse effect level“, or NOAEL, for inhibition of cerebral cholinesterase activity in rats [63]) for the CPF groups. Chicory inulin was dissolved in water at a concentration of 10 g/L. Animals were given access to the inulin-enriched or regular drinking water ad libitum. The treatments associated with each group is detailed in Figure 1.

Females were fed a standard diet and the corresponding gavage and drinking water for four consecutive months before mating them throughout gestation and lactation. Male rats were housed under the same conditions as females and received a standard diet. Gestation was assessed daily after mating (two females per male) by the presence of sperm in vaginal smears. The dams were then housed individually until birth and with their pups until weaning. The day of parturition was considered postnatal day 1 (PND1). The average litter size was 6 pups per dam. At the time of weaning (PND21), the female offspring were separated from their mother and housed with their littermates. Male offspring were included in a separate project [64,65]. They were housed according to density requirement in the EU legislation and fed a standard diet until the end of the experiment on PND60. Food and water consumption were measured. Dams were weighted daily throughout the experiment. To determine whether or not exposure during gestation and lactation induced growth retardation, pups were weighed at birth (PND3), at weaning (PND21) and at PND60.

At the end of the experiment (PND21 for dams, PND60 for offspring), the rats were euthanized by intraperitoneal administration of sodium pentobarbital, and plasma as well as various intestinal segments (ileum, colon and cecum) and internal organs (spleen, liver, mesenteric fat tissue and gonadal fat tissue) were removed under sterile conditions.

### 2.2. Metabolic Perturbations

Approximately 3 mL of intracardiac blood was collected from each animal into a test tube and centrifuged at 1500× *g* for 10 min at 4 °C. The harvested serum samples were transferred to clean test tubes before analyses.

Standard spectrophotometric methods based on an automation program from the University Hospital of Amiens (KT-6400 analyzer (Genrui Biotech Inc., Shenzhen, China)) were used to measure the following serum parameters: Cholesterol, High Density Lipoprotein (HDL), Low Density Lipoprotein (LDL), triglycerides (TG), blood sugar.

### 2.3. Disruption of Key Bacteria

#### 2.3.1. Concentrations of Selected Intestinal Bacteria and Bacterial Translocation

Organs were weighed, placed in a sterile stomacher bag and homogenized in Ringer’s saline solution (Bio-Rad, Marnes-la-Coquette, France) before serial 1/10 dilutions. Then, 1 mL of homogenate of intestinal segments and sterile organs was spread on different selective and non-selective media for qualitative and quantitative cultures of selected aerobic and anaerobic intestinal bacteria (Bactron Anaerobic, Sheldon Manufacturing, Cornelius, OR, USA) and incubated at 37 °C for 48 h to 4 days [51,52]. After incubation, bacteria were identified using standard microbiological techniques [45,50,66]. The isolated colonies grown on Petri dishes were counted using an automatic colony counter (Scan^®^ 500, Interscience, St Nom la Bretèche, France) and expressed in log (CFU)/g of tissue.

#### 2.3.2. Microbial Metabolites

Primary dilution samples for selected gut-bacteria analyses were used for short chain fatty acid (SCFA) measures. The supernatant was acidified to pH 2 using 25 µL of H_2_SO_4_ (2 M) and injected into a BP21 gas chromatography (GC) column (length: 30 m, inner diameter: 0.53 µm, film thickness: 1 µm) with an internal standard (4-hydroxy-4-methyl-2-pentanone). H_2_ was used as a carrier gas at a flow rate of 1.5 mL/min. The initial oven temperature was 135 °C and was held there for 6 min, then raised by 25 °C/min to 180 °C and held for 1 min, then increased by 25 °C/min to 230 °C, and finally maintained at 230 °C for 1 min. Glass liner ultra-inert was used for the split injection. The temperatures of the flame ionization detector and the injection port were 280 °C and 240 °C, respectively. The flow rates of H_2_ and air as makeup gas were 40 and 400 mL/min, respectively. The sample volume injected for the GC analysis (AutoSystem XL; PerkinElmer) was 1 µL and the run time for each analysis was approximately 10 min. The three main short-chain fatty acids (acetic acid, propionic acid, butyric acid) were identified as a function of the retention time of the different elution peaks. The quantification was obtained by comparison with a standard curve [67].

#### 2.3.3. Serum Lipopolysaccharide (LPS)

Plasma LPS is a useful marker for the identification of increased intestinal permeability and thus of intestinal injury. Plasma assay was performed with the Rat Lipopolysaccharide ELISA kit according to the manufacturer instructions (#CSB E14247r, CliniSciences, Nanterre, France).

### 2.4. Statistical Analyses

Statistical analyses were performed with StatView software (version 5.0, Abacus Concepts Inc., Berkeley, CA, USA) and SPSS Statistics software (version 25.0). Data were analyzed using one-way and two-way ANOVA and KHI2 assay. The independence of endpoints measured in littermates was evaluated by one-way ANOVA for each parameter and treatment group to assess intra and intergroup effects. Except for body weight at PND3 and PND21 in control group, litter effect was not detected. Offspring were thus considered a valid experimental unit for the F1 impact analyses. In all analyses, the threshold of statistical significance was set at *p* < 0.05.

## 3. Results

### 3.1. Animal Weight and Weight Gain

**Figure 2:**
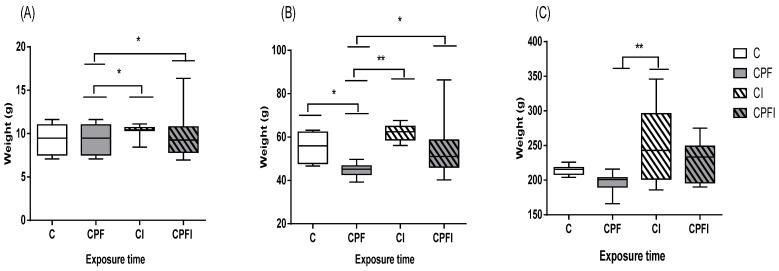
Effects of perigestational exposure to CPF and inulin on the body weight of the offspring of female rats 3 days after birth (PND3, (**A**)), as juveniles (PND21, (**B**)) and young adults (PND60, (**C**)). Values are expressed as mean ± SEM (*n* = 7–10) using analysis of variance (ANOVA) and Tukey’s test. Significance * *p* < 0.05; ** *p* < 0.01; C: Control; CPF: Chlorpyrifos; CI: Control inulin; CPFI: Chlorpyrifos + inulin; PND: post-natal day.

To mimic chronic exposure, we exposed dams for 4 months before gestation, as well as during gestation and lactation (Table 1). No significant differences for weight gain were observed in dams. At PND3 (Figure 2A) the pups from dams exposed to CPF alone (7.5 g) were significantly smaller than the corresponding inulin group (9.9 g for CPFI, *p* = 0.043). This was still significant at weaning (45.3 g for CPF vs. 55.2 g for C and 56.0 g for CPFI, *p* = 0.042 and *p* = 0.050, respectively, Figure 2B). No significant variation remained at early adulthood (PND60, Figure 2C). To complete these observations, no variation was observed in drinking and eating patterns either.

### 3.2. Metabolic Perturbations

**Figure 3:**
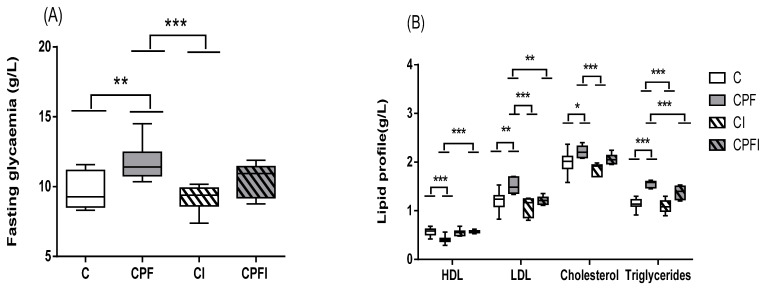
Effects of perigestational exposure to CPF and inulin on blood glucose (**A**) and lipid levels (**B**) in female offspring. Blood glucose, Total cholesterol (TC), plasma triglycerides (TG), high-density lipoproteins (HDL) or low-density lipoproteins (LDL) were measured in plasma. Values are expressed as mean ± SEM (*n* = 7–10) using analysis of variance (ANOVA) and Tukey’s test. Significance * *p* < 0.05; ** *p* < 0.01, *** *p* < 0.001. C: Control; CPF: Chlorpyrifos; CI: Control inulin; CPFI: Chlorpyrifos + inulin.

In dams and offspring to PND60, chronic exposure to CPF significantly altered the glycemic and lipid profile in rats (Table 2 and Figure 3).

Blood fasting glucose was significantly higher in dams exposed to CPF compared to the control (*p* = 0.0001) and the corresponding inulin-fed animals (*p* = 0.004), but only versus control group in offspring (*p* = 0.002, Figure 3A). Following direct exposure to CPF in dams or indirectly via lactation, lipid balance was significantly disturbed. In dams treated with CPF the total cholesterol increased by 20% (*p* = 0.05) and 28% for triglycerides (*p* = 0.029), while HDL-cholesterol levels decreased by 33% (*p* = 0.048). Moreover, in female offspring, as shown in Figure 3B, the CPF effect at PND60 remained strong with total cholesterol increased by 16% (*p* = 0.012), triglycerides by up to 15% (*p* = 0.0001 and *p* = 0.009 against C and CPFI, respectively), and LDL levels by up to 27% (*p* = 0.001 and *p* = 0.002 against C and CPFI, respectively). Finally, HDL levels decreased up to 30% in plasma of CPF offspring (*p* = 0.0001 and *p* = 0.0001 against C and CPFI, respectively).

### 3.3. Disturbances of the Selected Intestinal Bacteria

#### 3.3.1. Concentrations of Selected Intestinal Bacteria

**Figure 4:**
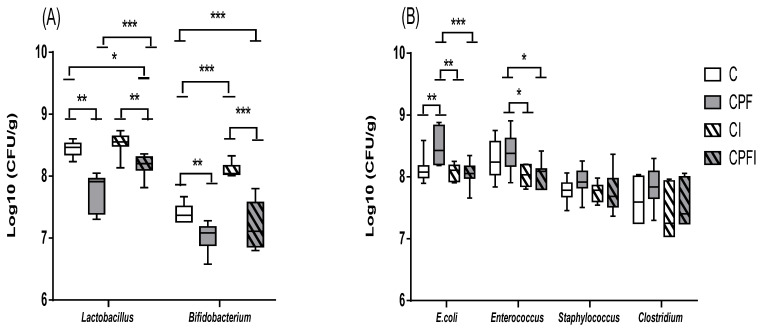
Effects of perigestational exposure to CPF and inulin on beneficial flora (**A**) and on potentially pathogenic flora (**B**) of female offspring. Values are expressed as mean ± SEM (*n* = 7–10) using analysis of variance (ANOVA) and Tukey’s test. Significance * *p* < 0.05; ** *p* < 0.01, *** *p* < 0.001. C: Control; CPF: Chlorpyrifos; CI: Control inulin; CPFI: Chlorpyrifos + inulin.

The pesticide altered the levels of selected gut bacteria in the dams. With regards to the total flora (total aerobic flora *p* = 0.673, total anaerobic flora *p* = 0.673), no significant difference between the groups was observed. When investigating specific bacterial populations (Table 3 and Table 4), the concentration of *Lactobacillus* spp. decreases significantly by 1 log in the dams treated with CPF compared to the controls (*p* = 0.05). For *Bifidobacterium* spp., the results revealed no significant difference among the groups, but the level of *Bifidobacterium* decreased in the groups treated with CPF (0.3 log vs. control group). When considering potentially pathogenic flora, bacterial populations were more abundant for animals treated with CPF (0.4 log; *p* = 0.009 and 0.7 log; *p* = 0.024) against control group for *E. coli* and *Enterococcus*, respectively. Moreover, that the addition of the prebiotic decreased by 1 log the abundance of *Enterococcus* (*p* = 0.003). *Clostridium* population was increased in the CPF group (0.4 log vs. control) but the results showed no significant difference among the groups. In the offspring, no significant difference among the groups was observed for the total flora. The potentially beneficial flora, *Lactobacillus* spp. and *Bifidobacterium*, was significantly modulated in the different treatment groups (Figure 4A). Specifically, the level of *Lactobacillus* spp. decreased by 0.7 log with CPF against the control group (*p* = 0.0001) while inulin showed a protective 0.5 log increase in bacterial content for *Lactobacillus* (*p* = 0.0001). For potentially pathogenic flora (Figure 4B), *E. coli* was significantly more abundant (0.4 log) in rats treated with CPF (*p* = 0.001 vs. control group) and decreased by 0.5 log in the CPF group with inulin supplementation *p* = 0.0001 vs. CPF). Similarly, inulin reduced by 0.4 log the number of *Enterococcus* found in the CPF group (*p* = 0.022). The results of *Staphylococcus* and *Clostridium* showed no significant difference among the groups, but their abundance tended to increase with exposure to pesticides (0.2 log vs. control) and decrease with inulin supplementation (0.3 log vs. CPF only).

#### 3.3.2. Bacterial Metabolites

Another consequence of dysbiosis is the impaired production of short chain fatty acids (SCFA), which are dependent on various factors in the host microbiota. The profiles of individual SCFA differed depending on the treatment. The presence of CPF alone increased the acetic acid content (2%) in the dams, but not significantly. In the female offspring the CPF significantly increased the ratio of acetic acid and inulin prevented it (*p* = 0.012; CPF = 71.3% versus CPFI = 53.7%). The presence of inulin increased the levels of propionic and butyric acid in dams and offspring; variations were not significant for dams for either SCFA. In offspring, propionic acid was decreased with CPF and recovered with inulin (CPFI = 15.3% versus C = 15.4% versus CPF = 12.6%). Butyric acid inhibition by CPF and recovery by inulin, however, was significant in the offspring (*p* = 0.008; CPFI = 27.4% versus CPF = 16.0%).

#### 3.3.3. Bacterial Translocation

Overall, a large aerobic and anaerobic translocation to the spleen was observed in the CPF groups (33% of dams and 25% of offspring), compared to the control and control inulin group (0% of dams and offspring) and CPF inulin (50% of dams and 0% of offspring). The CPF versus control differences were statistically significant only in the female offspring (*p* = 0.046). These results were corroborated by serum LPS concentrations in dams which were two-fold higher for the CPF group compared to the control and inulin, but no significant difference was observed. This was not observed in the offspring.

## 4. Discussion

Overall, whether it is through direct or indirect exposure to OP, there are changes in body weight, disruption of lipid metabolism and glucose metabolism, and changes in levels of selected gut bacteria. However, how CPF disrupts these metabolisms is not fully understood.

### 4.1. Animal Weight and Weight Gain

Weight gain for the dams as well as birth weight have been investigated extensively with regards to risk factors of chronic disorders (obesity, type 2 diabetes), but also as an indicator of the toxic capacity during gestation due to the development of the offspring. Several in vivo studies have suggested that CPF treatment can increase or decrease body weight and birth weight, depending on the dose tested.

The results of the present study revealed that the body weight of the CPF-treated groups was significantly lower than that of the other groups after chronic exposure to low levels of CPF. This is consistent with reports by several authors [68,69,70,71,72]. Researchers have suggested that a decrease in body weight may be due to increased oxidative stress and the degeneration of lipids and proteins [39,73,74]. Exposure to the 1 mg/kg dose did not significantly affect maternal weight gain compared to other groups. And the developmental assessment of female offspring indicated that all CPF-treated groups had low body mass gain compared to other PND60 groups, which is consistent with previous findings [38] and other studies [37,68,75,76].

### 4.2. Metabolic Perturbations

In this study, blood glucose concentration was markedly increased in both dams and female offspring. Consistent with these results, OP have been shown to induce hyperglycemia [37,38,77,78,79,80]. An increasing number of researchers have begun to focus on the mechanism of exposure to OP and hyperglycemia while other studies have reported that OP do not affect metabolic indices [35,81,82].

Changes in lipid profile due to CPF administration were observed in this study, other studies also showed an increase in TG in agreement with the present results [41,83] while others have observed lower or unchanged TG levels [37,84].

CPF also disrupts serum TC levels. In our study, cholesterol levels were higher, and several studies are in agreement with our results [28,37,41,83,84]. They suggested that serum TC levels may be increased in the groups treated with CPF due to blockage of the hepatic bile ducts, resulting in decreased or stopped TC secretion in the duodenum [39,85].

The result of the present study also demonstrated the dyslipidemic effect exerted by chronic administration of low levels of CPF. This was evident by the increased LDL levels and decreased HDL levels recorded in the serum of CPF-exposed rats. Some authors have suggested that CPF causes liver damage and lipoprotein synthesis [28,41,83,84].

In our experiment, chronic exposure to low doses of CPF was also responsible for developmental alterations in male pups, characterized by low birth weight, decreased plasma growth factor (IGF1) levels, leptin and a small increase in insulinemia. These effects were still present at weaning but disappeared in early adulthood when the animals were no longer exposed [64].

### 4.3. Disturbances of the Selected Intestinal Bacteria

To summarize, levels of key bacteria were unstable in dams and offspring with an increase in potentially pathogenic flora to the detriment of potentially beneficial flora in rats treated with CPF. CPF exposure was associated with significant microbial perturbation, showing the influence of CPF exposure on a number of bacteria, with reduced abundance of *Lactobacillus* spp. and *Bifidobacterium* spp., and a higher level of *Enterococcus* spp., *E. coli*, *Staphylococcus* spp. and *Clostridium* spp. in rats treated with CPF. Similar results were shown in previous work and other studies [50,84,86,87,88,89]. These results demonstrate that exposure to CPF induces disturbances in the gut microbiota and that early gut microbiota disruption could lead to long-term effects.

Another consequence of dysbiosis is the altered production of short-chain fatty acids (SCFA), which depend on various factors in the host microbiota. Butyrate, propionate and acetate represent 90 to 95% of SCFA present in the colon [90,91]. They are produced by fermentation of dietary fibers and are often associated with the prevention of several pathologies linked to inflammation or oxidative stress [92]. These results are in agreement with previous results on CPF [38] and other results on the different pesticides carbendazim [93], permethrin [94] and propamocarb [95], which suggests that the perturbation of the intestinal microbiota following pesticide exposure altered the proportions of SCFA, disturbed the energetic homeostasis and elicited multiple tissue inflammatory responses.

The bacterial translocation was predominantly higher in the CPF groups than in the other groups. It is recognized that microbial dysbiosis exerts a strong influence on the intestinal permeability [45,96,97]. The translocation phenomenon could be explained by the increase in permeability observed after exposure to CPF [45,50]. CPF alone inhibits the expression of tight junction and structural genes, while inulin and CPF/inulin co-treatment tended to increase expression [54]. Neonatal translocation is completely normal and even essential for the maturation of the immune system [98], but its persistence after weaning could become pathogenic. It is, therefore, likely that the pesticide disrupted the tight junctions, while the mixture enhanced the activity of the tight junctions [54]. Higher LPS concentrations in the serum of dams is a marker of permeability as well as a risk factor for low grade inflammation [99]. These are also consistent with C-reactive protein (CRP) serum concentrations in dams following CPF exposure (data not shown) as a sign of inflammation.

### 4.4. Nutritional Prevention

Prebiotic dietary fibers are likely to selectively modulate the gut microbiota and exert positive health effects [100]. The prevention approach used in this study was based on the use of inulin, acting as a prebiotic to counteract the effects of CPF on the gut microbiota [38,54], which in turn affects intestinal functions, such as metabolism and the integrity of the intestine [101]. Co-treatment with inulin prevents dysbiosis as well as early markers of dysmetabolism in rats chronically exposed to CPF. However, we could not find any other work in the literature studying this particular prevention strategy to counter pesticides except for an article on zebrafish [102].

Present results indicated that inulin consumption improved the lipid and glycemic profiles, consistent with other studies. The mechanism by which inulin acts on glucose and lipid metabolism remains unclear. A number of hypotheses have been proposed regarding the effect of prebiotics on improving dysmetabolism. One mechanism included decreased absorption of cholesterol via intestinal epithelial cells [103]. Inulin is a soluble, viscous compound that increases the thickness of the unstirred layer of the small intestine, thereby inhibiting absorption of cholesterol [104]. Inulin does not bind to bile acid in the upper digestive tract; however, it can help soluble bile acids bind to bacteria or insoluble compounds, such as calcium phosphate, by lowering the pH of the cecum [105]. As a result, fecal excretion of bile acids increases cholesterol utilization to rebuild bile acid in the liver and decrease the concentration of circulating and hepatic cholesterol [106]. Also, inulin treatment acts on glucose metabolism by improving serum GLP-1 levels to suppress IL-6 secretion and production, and hepatic gluconeogenesis and resulted in moderation of insulin tolerance. These results indicate that intestine-liver crosstalk is the primary mechanism for moderating insulin resistance by inulin [106,107,108].

Specific changes in the composition of the gut microbiota occurred in the prebiotic group; inulin increased the number of *Lactobacillus* and *Bifidobacterium* but also reduced the number of *Enterococcus* spp., *E. coli*, *Staphylococcus* spp. and *Clostridium* spp. The combination of CPF and inulin reduced the number of enterococci, while the numbers of *Bifidobacterium* and *Lactobacillus* increased. Another potential mechanistic pathway may be behind the change in the composition of the gut microbiota after inulin supplementation leading to an increase in SCFA and decrease in cecal pH. SCFA are important for health because they improve energy metabolism in the liver and muscles and immune function in the large intestine [109,110,111,112].

### 4.5. Perigestational Modulation of CPF

In the general population, new parents tend to look more closely into the food given to their infants, trying to aim for organic, pesticide-free options, even though their own diet may not be modified dramatically. It is not uncommon either that the diet of prospecting parents does not differ from that of the global population [113,114,115]. With this in mind, we set up this model to expose the dams to treatments before mating and continue it up to weaning but discontinue treatment for the offspring.

Differences in the toxicity of CPF between fetus and mother have already been reported due to the lipophilic power of CPF, which can cross the placental barrier [28,68,76,116]. CPF and its metabolite DETP (diethyl thiophosphonate) have been studied and published in the literature and residues have been detected in newborns [116,117]. 3,5,6-trichloro-2-pyridinol (TCPy), a specific metabolite of ethyl and methyl chlorpyrifos, was also recently found in the hair collected at birth in 311 new mothers [118]. This is, therefore, a critical window of exposure to toxic environment xenobiotics through aerosol or oral intake. During gestation and early childhood, the internal organs are still undergoing a process of maturation. Previous work showed that the intestine and microbiota maturation were delayed following CPF treatment [50]. The objective of the work presented herein was to better understand whether these perturbations would still have consequences if treatment was interrupted after weaning.

These data, together with the knowledge that a number of chronic disorders such as obesity or type 2 diabetes can be triggered by in utero imprinting, suggest that it is likely that CPF may lead to resilient effects event after treatment was discontinued. The results presented here for the offspring clearly support this hypothesis. It would be interesting to see if there were some epigenetic modifications in target tissues. Unfortunately sampling conditions did not allow for genomic analyses.

In addition, birth weight is thought to be predictive of chronic disorders. A low birth weight, often observed in experiments with OP, tends to be associated with adult risks of obesity and metabolic syndrome, whilst a larger birth weight would more likely be associated with increased risks of type 2 diabetes. Data show that both glucose and lipid profiles are modified by CPF, more targeted analyses or long-term experiments would be required to clearly differentiate between the two pathologies as they encounter a number of overlapping features.

## 5. Conclusions

The study allowed us to gather new evidence of resilient and indirect impact on offspring of CPF in metabolic disorders as summarized in Table 5. Modulations in specific bacterial populations as well as glucose and lipid profiles suggest mechanisms of dysmetabolism typical of obesity and diabetes [58]. The large number of endpoints measured would require statistical partitioning among variables, and this is a bias that may need to be highlighted when reaching overall conclusions. The strength of this work lies in the duration of the exposure of dams to the pesticide and the measure of indirect impact to the offspring to better mimic real-life settings. Further investigation in the epigenetic imprinting that could be associated with transgenerational effects would be complementary to these findings. In addition, while cultured microbiology may seem to be outdated for microbiota analyses compared to shotgun metagenomics, it is of great interest to follow up on bacterial translocation and perturbation of the intestinal permeability for a complete picture of the intestinal ecosystem. Finally, the nutritional prevention strategy can be further explored using other prebiotic molecules, probiotics or even testing different windows of exposure. A high-energy diet is also a risk factor leading to similar perturbations. It would be interesting to see whether mechanisms with CPF or high-fat diets are similar, and if a mix of dietary and environment factors would have synergistic effects. This hypothesis has been tested, samples are being analyzed and results are underway.

## Figures and Tables

**Table 1:** Body weight (g) before gestation (1st month and 4th month), during gestation (21st day of gestation) and during lactation (21st day of lactation) of dams exposed to CPF and inulin. The values are expressed as mean ± SEM (*n* = 4). Significantly different (*p* < 0.05) using analysis of variance (ANOVA) and Tukey’s test. C: Control; CPF: Chlorpyrifos; CI: Control inulin; CPFI: Chlorpyrifos + inulin.

	Before Gestation (g)				Gestation (g)		Lactation(g)	
	1st Month		4th Month		21st Day of Pregnancy		21st Day of Lactation	
Control	268.7 ± 28.28	F value = 0.980 *p* value = 0.44	308.7 ± 31.75	F value = 1.289 *p* value = 0.32	396.6 ± 88.79	F value = 0.347 *p* value = 0.793	334.6 ± 17.47	F value = 3.140 *p* value = 0.096
CPF	253.5 ± 23.86		286.7 ± 30.20		413.5 ± 105.35		311.0 ± 19.89	
Control + Inulin	260.7 ± 33.62		297.2 ± 35.77		443.0 ± 68.06		392.0 ± 56.56	
CPFI	283.0 ± 10.42		325.0 ± 11.63		447.0 ± 46.61		390.7 ± 42.46	

**Table 2:** Effects of exposure to CPF and inulin on blood sugar (A) and lipid (B) levels in dams. Blood glucose, total cholesterol (TC), plasma triglycerides (TG), high density lipoproteins (HDL) or low density lipoproteins (LDL) were measured in plasma. The values are expressed as mean ± SEM (*n* = 4). Significantly different (*p* < 0.05) using analysis of variance (ANOVA) and Tukey’s test. C: Control; CPF: Chlorpyrifos; CI: Control inulin; CPFI: Chlorpyrifos + inulin.

	Glucose (g/L)		Cholesterol (g/L)		Triglycerides (g/L)		HDL (g/L)		LDL (g/L)	
Control	7.8 ± 0.83	F value = 40.018 *p* value = 0.0001	1.7 ± 0.25	F value = 6.480 *p* value = 0.02	1.4 ± 0.02	F value = 8.573 *p* value = 0.01	0.6 ± 0.02	F value = 3.817 *p* value = 0.066	0.8 ± 0.27	F value = 5.549 *p* value = 0.029
CPF	11.6 ± 0.08		2.4 ± 0.24		1.8 ± 0.15		0.4 ± 0.08		1.6 ± 0.32	
Control + inulin	7.4 ± 0.15		1.4 ± 0.17		1.3 ± 0.12		0.5 ± 0.10		0.5 ± 0.25	
CPFI	9.4 ± 0.40		2.0 ± 0.39		1.6 ± 0.12		0.6 ± 0.04		1.0 ± 0.36	

**Table 3:** Effects of exposure to CPF and inulin on beneficial flora in dams. The values are expressed as mean ± SEM (*n* = 4). Significantly different (*p* < 0.05) using analysis of variance (ANOVA) and Tukey’s test. C: Control; CPF: Chlorpyrifos; CI: Control inulin; CPFI: Chlorpyrifos + inulin.

	Beneficial Flora (CFU/g)			
	*Lactobacillus*		*Bifidobacterium*	
Control	8.2 ± 0.09	F value = 4.672 *p* value = 0.04	7.2 ± 0.61	F value = 2.192 *p* value = 0.177
CPF	7.2 ± 0.22		6.5 ± 0.32	
Control + inulin	8.3 ± 0.66		7.6 ± 0.53	
CPFI	7.9 ± 0.48		7.0 ± 0.60	

**Table 4:** Effects of exposure to CPF and inulin on potentially pathogenic flora in dams. The values are expressed as mean ± SEM (*n* = 4). Significantly different (*p* < 0.05) using analysis of variance (ANOVA) and Tukey’s test. C: Control; CPF: Chlorpyrifos; CI: Control inulin; CPFI: Chlorpyrifos + inulin.

	Potentially Pathogenic Flora (CFU/g)							
	*E.coli*		*Enterococcus*		*Staphylococcus*		*Clostridium*	
Control	7.9 ± 0.26	F value = 7.662 *p* value = 0.013	7.6 ± 0.10	F value = 13.012 *p* value = 0.003	7.3 ± 0.28	F value = 3.293 *p* value = 0.088	6.7 ± 0.35	F value = 0.935 *p* value = 0.473
CPF	8.3 ± 0.04		8.3 ± 0.14		8.2 ± 0.10		7.1 ± 0.20	
Control + inulin	7.4 ± 0.31		7.4 ± 0.03		7.2 ± 0.28		7.0 ± 0.17	
CPFI	7.9 ± 0.19		7.3 ± 0.33		7.5 ± 0.70		7.0 ± 0.36	

**Table 5:** Summary of direct and indirect impact of CPF and protective impact of inulin co-exposure.

		Direct Effect (Dams)		Indirect Effect (Offspring)	
		CPF	Inulin on CPF	CPF	Inulin on CPF
Metabolic	Weight	-	-	Loss (at PND21 only)	Recovery
	Glucose	Increased	Recovery	Increased	-
	Cholesterol	Increased (total) Increased (LDL) Decreased (HDL)	Recovery (total) - (LDL) Recovery (HDL)	Increased (total) Increased (LDL) Decreased (HDL)	- (total) Recovery (LDL) Recovery (HDL)
	Triglycerides	Increased	-	Increased	Recovery
Bacterial	Selected bacteria (+)	Decreased (*Lactobacillus*) - (*Bifidobacterium*)	- (*Lactobacillus*) - (*Bifidobacterium*)	Decreased (*Lactobacillus*) Decreased (*Bifidobacterium*)	Recovery (Lactobacillus) - (*Bifidobacterium*)
	Selected bacteria (−)	Increased (*E. coli*) Increased (*Enterococcus*)	- (*E. coli*) Recovery (*Enterococcus*)	Increased (*E. coli*) Increased (*Enterococcus*)	Recovery (*E. coli*) Recovery (*Enterococcus*)
	Metabolic ratio	-	-	Increased Acetate Decreased Butyrate	Recovery
	Translocation	-	-	Increased	-

## Data Availability

No open access to study data.

## Abbreviations

B.W.	body weight
C	Control
ChE	choline esterase
CI	control + inulin
CNS	central nervous system
CPF	chlorpyrifos
CPFI	CPF + inulin
CRP	C-reactive protein
DETP	diethyl thiophosphonate
DOHaD	Developmental origin of Health and Disease
EPA	United States Environmental Protection Agency
GC	gas chromatography
GLP1	glucose-like protein-1
HDL	high-density lipoprotein
IGF1	insulin growth factor-1
IL6	interleukin-6
LDL	low-density lipoprotein
LPS	lipopolysaccharide
NOAEL	no observed adverse effect level
OP	organophosphate
PND	post-natal day
PNSE	National Health and Environment Plans
SCFA	short chain fatty acid
SEM	standard error of the mean
T2D	type 2 diabetes
TCPy	3,5,6-trichloro-2-pyridinol
TG	triglyceride
WHO	World Health Organization
